# An organizational readiness intervention and randomized controlled trial to test strategies for implementing substance use disorder treatment into primary care: SUMMIT study protocol

**DOI:** 10.1186/s13012-015-0256-7

**Published:** 2015-05-08

**Authors:** Allison J Ober, Katherine E Watkins, Sarah B Hunter, Karen Lamp, Mimi Lind, Claude M Setodji

**Affiliations:** RAND Corporation, 1776 Main Street, Santa Monica, CA 90407 USA; Venice Family Clinic, 604 Rose Avenue, Venice, CA 90291 USA

**Keywords:** Implementation, Organizational readiness, Evidence-based substance use disorder treatment, Primary care, Collaborative care, Care coordination, Medication-assisted treatment, Extended-release injectable naltrexone, Vivitrol, ®Buprenorphine/naloxone, Suboxone, ®Motivational interviewing

## Abstract

**Background:**

Millions of people who need treatment for substance use disorders (SUD) do not receive it. Evidence-based practices for treating SUD exist, and some are appropriate for delivery outside of specialty care settings. Primary care is an opportune setting in which to deliver SUD treatment because many individuals see their primary care providers at least once a year. Further, the Patient Protection and Affordable Care Act (PPACA) increases coverage for SUD treatment and is increasing the number of individuals seeking primary care services. In this article, we present the protocol for a study testing the effects of an organizational readiness and service delivery intervention on increasing the uptake of SUD treatment in primary care and on patient outcomes.

**Methods/design:**

In a randomized controlled trial, we test the combined effects of an organizational readiness intervention consisting of implementation tools and activities and an integrated collaborative care service delivery intervention based on the Chronic Care Model on service system (patient-centered care, utilization of substance use disorder treatment, utilization of health care services and adoption and sustainability of evidence-based practices) and patient (substance use, consequences of use, health and mental health, and satisfaction with care) outcomes. We also use a repeated measures design to test organizational changes throughout the study, such as acceptability, appropriateness and feasibility of the practices to providers, and provider intention to adopt the practices. We use provider focus groups, provider and patient surveys, and administrative data to measure outcomes.

**Discussion:**

The present study responds to critical gaps in health care services for people with substance use disorders, including the need for greater access to SUD treatment and greater uptake of evidence-based practices in primary care. We designed a multi-level study that combines implementation tools to increase organizational readiness to adopt and sustain evidence-based practices (EBPs) and tests the effectiveness of a service delivery intervention on service system and patient outcomes related to SUD services.

**Trial registration:**

Current controlled trials: NCT01810159

**Electronic supplementary material:**

The online version of this article (doi:10.1186/s13012-015-0256-7) contains supplementary material, which is available to authorized users.

## Background

Substance use disorders (SUD) continue to be under-identified and under-treated [1]. In 2013, 22.7 million people aged 12 or older needed treatment for an illicit drug or alcohol use problem; of these, 20.2 million did not receive it [1]. The consequences of untreated alcohol and drug abuse are great and include increased risk of disease, injury, disability, and death [2,3] as well as hundreds of billions of dollars in costs to the criminal justice, social welfare, and health care systems [4-6]. Historically, treatment of SUD has taken place in residential and outpatient specialty care settings. Although specialty care settings play an important role for individuals with severe dependence, long waiting lists, stigma, and the lack of public funding for patients without insurance coverage have contributed to the lack of access. Further, many people who need treatment are not aware that they need it, are not ready for treatment, or do not know how or where to seek treatment [7].

Primary care clinics are a feasible and opportune setting in which to identify and provide treatment to people with SUD. Studies suggest that the prevalence of alcohol use disorders and use of illicit drugs is higher among primary care and emergency room patients than it is in the general population [8,9]. Further, most individuals (82%) visit a health professional at least once a year, thus providing ample opportunity for providers to identify patients in need of treatment [10]. Research suggests that integrating SUD treatment and general health care can result in less utilization of inpatient care and fewer emergency room visits [11] and that integrated care is acceptable to patients with an SUD [12].

However, despite the potential benefits of providing SUD screening and treatment in primary care and the existence of evidence-based practices (EBP) suitable for delivery in these settings [13-21], uptake of evidence-based SUD treatments in primary care has been slow. Accordingly, patients are unlikely to receive treatment for their SUD in primary care [20-24]. Some of the organizational barriers to providing SUD treatment in primary care settings include lack of insurance reimbursement, perceived lack of time to fully assess and discuss substance use, and lack of administrative buy-in for integrating SUD care into medical practices [25,26]. At the physician level, perceived barriers to SUD treatment adoption include negative attitudes toward people with SUD, lack of confidence among physicians in their ability to treat SUDs, lack of adequate role models and access to decision support consultants, and deficiencies in training and expertise in addiction treatment [13,25-28].

Research on introducing new practices into health care and other organizations suggests that intervention at both the organizational level (i.e., to increase organizational readiness to adopt new practices) and service delivery system level (i.e., reorganizing how care is provided to support the new practice) may both be necessary to integrate and sustain EBP [29-31]. Organizational readiness refers to “the extent to which organizational members are psychologically and behaviorally prepared to implement organizational change” [32]. Interventions that increase an organization’s commitment to change and the ability of the members of the organization to visualize how the new practice could be adopted and incorporated into existing practices are both important to increasing organizational readiness and adoption of EBP [33]. However, even when an organization exhibits high organizational readiness, change may not be successful unless attention is paid to how the new practice is supported and integrated into existing care practices. Further, adapting new practices to fit the nuances of a setting is a key component of whether the practice is ultimately accepted and adopted. As Damschroder et al. note, “without adaptation, interventions usually come to a setting as a poor fit, resisted by individuals who will be affected by the intervention, and requiring an active process to engage individuals in order to accomplish implementation [30].”

To address the need for change at two levels—organizational and service delivery system—to increase the intent and ability of primary care providers to identify and treat opioid and alcohol use disorders (OAUD), we designed a multi-level study to create and evaluate change at both levels. We call this study substance use motivation medication integrated treatment (SUMMIT) and focus on alcohol and opiate use disorders because of their relevance to the clinic population and availability of effective medications. At the organizational level, we test the effects of an organizational readiness intervention on the organization’s readiness to identify and treat individuals with opioid and alcohol use disorders. At the service delivery system level, we use Wagner’s Chronic Care Model [34] to reorganize and guide how care for OAUD is provided and supported; we call the service delivery intervention integrated collaborative care (ICC). Integrated, collaborative approaches have been successful in improving outcomes for patients experiencing a variety of different chronic illnesses, including diabetes [35], asthma [35], and depression [36]. ICC has improved implementation of evidence-based treatments and quality of care [37], lowered costs [38], improved patient outcomes [39-42], and is thought to be feasible for and sustainable in primary care clinics [43]. We test the effects of the organizational readiness intervention using a repeated measures pre-post design and the impact of the service delivery intervention on patient-level outcomes using a randomized controlled trial (RCT) to compare the service delivery intervention (ICC) with “service as usual” (SAU) on service system and patient outcomes. We hypothesize that provider (providers include administrators, medical and mental health providers, and other staff) readiness to implement the EBP and patient-centered SUD care will improve from the pre-organizational readiness intervention period (year 1) to the post-readiness intervention periods (years 2–5); that patients in the ICC condition will report more integrated, patient-centered evidence-based care for their opioid and/or alcohol use disorders, will be more likely to receive OAUD care, and will have lower overall health care utilization (e.g., emergency department and medical visits) than patients in the SAU condition; and that provider adoption of EBP will increase from year 2 to years 3 and 4 and that providers will still be delivering OAUD EBP a year after completion of the study in year 5. We also hypothesize that patients in the ICC condition will report less substance use, fewer SUD consequences, higher health and mental health functioning, and greater satisfaction with their SUD care 6 months after enrollment than SAU patients.

The evidence-based practices that we are introducing into the clinic are two medications—buprenorphine/naloxone (BUP/NX) (trade name Suboxone®) for opioid dependence and extended-release injectable naltrexone (XR-NTX) (trade name Vivitrol®) for alcohol dependence—and a motivational interviewing (MI)-based behavioral treatment for those with abuse or dependence of either substance. BUP/NX has been proven effective for patients with opioid (heroin as well as prescription opioid) dependence and is feasible for delivery in office-based settings [12,44-49]; XR-NTX has been found effective for people with alcohol or opioid dependence and also is feasible for delivery in primary care [50-54]. Due to greater complexity for administration for opiate dependence, in this study, XR-NTX is used only to treat alcohol dependence. MI-based interventions have improved SUD treatment outcomes [18,19,55,56].

In this article, we present our methods, including study setting; conceptual framework; study design; participant recruitment; a description of the interventions, which consist of the organizational readiness intervention and the service delivery intervention; as well as our measures, data collection procedures, and analysis plan. We conclude with a discussion of the study’s unique design and its relevance to implementation of OAUD treatment in primary care, and we note the study’s limitations.

## Methods/design

### Study setting

We are conducting the study in a large urban, federally qualified health center (FQHC) in Los Angeles that serves approximately 20,000 patients annually. The study is taking place at the FQHC’s two largest sites. We elected to hold the study in an FQHC because of the expected influx of patients into publicly funded clinics due to expanded coverage, an increased funding and an increase in the number of clinics due to the Patient Protection and Affordable Care Act (PPACA) [57], and the greater opportunity to reach more individuals who need treatment. The clinic currently has integrated mental health services and provides treatment for anxiety and depressive disorders; however, prior to the study, the clinic did not conduct any screening or treatment for SUD. If substance misuse was identified, patients were sometimes, but not systematically, referred to specialty care.

### Conceptual framework

**Figure 1:**
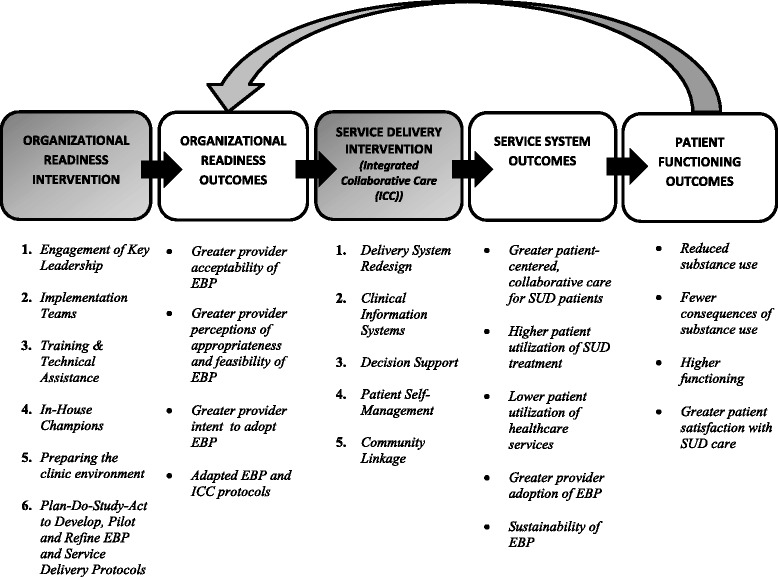
SUMMIT conceptual framework for integrating SUD EBP into primary care.

In our conceptual framework, illustrated in Figure 1, the organizational readiness intervention increases provider readiness to use each of the EBP for OAUD (the two medications and the MI-based behavioral therapy) as well as readiness to adopt ICC (the service delivery intervention) to deliver the three EBP. The organizational readiness intervention consists of well-studied “implementation” tools, designed to increase the readiness of an organization to implement and deliver new practices. Our measures of organizational readiness are provider acceptability of the EBP, provider perceptions of EBP appropriateness and feasibility, and provider intention to adopt each EBP. In addition, because an aspect of organizational readiness is the ability of providers to visualize how the new practices can be adopted and integrated into the existing workflow [33], a final measure of readiness is the development of locally tailored EBP protocols and an ICC protocol that shows how the EBP will fit into clinic workflow.

In the second part of the conceptual framework, the service delivery intervention (ICC) facilitates the uptake of the EBP and affects service system outcomes (e.g., patient-centered SUD care, measured at the level of the patient and provider, and service utilization, measured at the level of the patient) and patient functioning outcomes (e.g., substance use, consequences of use, both measured at the level of the patient). While organizational readiness may improve immediately following the organizational readiness intervention, we expect that once the three EBP are implemented through ICC and the staff sees improved patient outcomes, a feedback loop will occur, leading to even greater staff acceptance of the new practices.

### Study design

**Figure 2:**
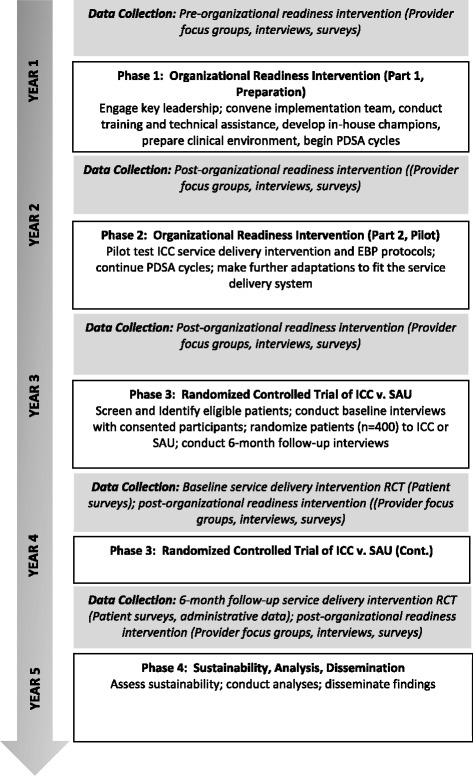
SUMMIT study design and timeline.

The study is designed to test the combined effect of both an organizational readiness intervention (which includes a 1-year organizational preparation period and an 8-month pilot of the ICC condition study) and a service delivery intervention (see Figure 2). We examine the effects of the interventions on organizational readiness, service system, and patient outcomes, all of which are believed to be important in understanding the uptake of new practices [58]. To test the unique effects of the organizational readiness intervention on provider outcomes, we use a pre-post-intervention design and measure these outcomes at the beginning of the study and then again at the end of year 1. At the end of year 1, we implement an eight-month pilot test of ICC. During the pilot test, providers gain experience with the protocols, and the protocols are iteratively adapted and refined based on provider feedback. Because we hypothesize that readiness outcomes may continue to improve as providers gain experience with ICC and the three treatments, we continue to measure organizational readiness outcomes annually at years 2–5. Thus, changes in outcomes between year 1 and years 2–5 reflect the combined effect of both the organizational readiness intervention and ICC on provider outcomes. To test the effects of the interventions on service system and patient-level outcomes, we are conducting an RCT to compare the effects of ICC with SAU. Service-system outcomes are patient-centered collaborative care, utilization of SUD treatment, patient utilization of health care services, provider adoption of EBP, and sustainability of EBP. Patient-level outcomes include substance use, consequences of use, physical and mental health functioning, and patient satisfaction with SUD care.

For the RCT, all patients are screened for drug and alcohol use by clinic staff as part of usual care; eligible consenting patients (i.e., those with risky use or worse) are referred for further eligibility screening by the research staff, and eligible patients (*N* = 400) are invited to participate in the study. After consenting and completing the baseline interview at one of the study sites, patients are randomized to the ICC or SAU study condition. We use a concealed randomization protocol so neither patient nor research staff is aware of the randomization until after the baseline interview is completed when research staff open sequentially numbered envelopes that contain the randomization assignment. Assignments were made in advance by a statistician using R software. Patients complete a follow-up interview by telephone 6 months after the baseline interview.

The design is a variation of a “hybrid type 2” study, which Curran et al. [59] describe as the “simultaneous testing of a clinical intervention and an implementation intervention/strategy.” In this case, the organizational readiness intervention is the implementation intervention/strategy and the ICC service delivery intervention is the clinical intervention. The design, which incorporates implementation outcomes such as intention to adopt EBP as well as service system and patient outcomes, follows the recommendations for implementation research outcomes suggested by Proctor et al. [58].

### Study participants

#### Organizational readiness intervention

Organizational readiness intervention participants are full-time clinic administrators, medical and mental health providers, and other clinic staff, including medical assistants, discharge coordinators, and front desk and security staff who agree to participate in interviews, focus groups and/or surveys (*N* = 70).

#### Service delivery intervention

Service delivery intervention participants are full-time medical and mental health providers (not including residents) as well as patients who come to the clinic for a medical visit; the participants initially screen positive for risky (or worse) alcohol or opioid use using an adapted NIDA Quick Screen [60] and then meet all study eligibility criteria and consent to participate in the study (*N* = 400). To be eligible for the study, patients must be 18 years or older; meet the “Diagnostic and Statistical Manual of Mental Disorders, fourth edition (DSM IV)” criteria for abuse of or dependence on alcohol or opioids (heroin or prescription opioids) (assessed using the WHO ASSIST [61]); must not have marked functional impairment from bipolar disorder or schizophrenia; speak English or Spanish; and are not currently in treatment for SUD.

### Interventions

#### Organizational readiness intervention

To create organizational readiness to provide evidence-based treatment for OAUD, we employ multiple tools and activities known to facilitate adoption of EBP as follows: (1) engaging (and obtaining buy-in from) key administrators through regular administrator and board briefings about the proposed study and how to best prepare the organization to implement the ICC intervention and providers to adopt the EBP [29,62]; (2) convening an implementation team that includes key clinical leadership to develop the ICC service delivery intervention and EBP protocols that fit the clinic [29,63]; (3) selecting and training physician and therapist champions to serve as role models for adopting the EBPs [29]; (4) providing trainings for the staff and providers on the ICC intervention and evidence-based treatment for opioid and alcohol use disorders [64,65]; and (5) preparing the clinic environment to identify patients with SUD by instituting universal screening and brief intervention procedures. After preparing the organization, we then conduct the final part of the organizational readiness intervention—piloting the EBP and ICC protocols and making iterative adaptations using plan-do-study-act (PDSA) cycles [66,67]. PDSA cycles offer a structured approach to engaging staff in making iterative, feedback-based changes in service delivery [65,66].

#### Service delivery intervention

We use Wagner’s Chronic Care Model (CCM) [35] as the theoretical basis for the service delivery intervention (ICC). The ICC intervention is comprised of five components that have been shown to result in improved patient outcomes; each component supports the delivery of the planned care for opioid and alcohol use disorders: (1) redesigning the delivery system to support the delivery of the EBP and establishing a care coordinator; (2) modifying clinical information systems to provide alerts to indicate that patients have problematic substance use and developing a patient registry used by the care coordinators to monitor and track patients; (3) providing expert consultation to therapists for complex cases; (4) offering patients self-management tools; and (5) identifying and establishing linkages with community resources. These components are thought to lead to productive patient-provider interactions, which, in turn, lead to improved service system and patient outcomes. The ICC components are described in greater detail in Additional file 1.

### Outcomes, procedures, measures, and analysis plan

Next we describe our data collection procedures, measures, and analysis plans for the organizational readiness and service delivery interventions.

#### Organizational readiness intervention

##### Procedures

We use qualitative and quantitative methods to study our organizational readiness outcomes as follows: 1) *Provider focus groups and semi-structured interviews*. We conduct focus groups with medical and mental health providers and one-on-one interviews with key administrators to inform the development of our intervention and EBP protocols and to understand perceptions of acceptability, appropriateness, feasibility, and intention to adopt the ICC and EBP protocols. For both the focus groups and the interviews, we follow a semi-structured protocol guide that asks “grand tour” questions related to each domain (i.e., general thoughts about ICC and the EBP), and includes specific probes for more detailed responses. 2) *Provider Surveys*. We also conduct surveys in years 1–5 with all the staff and providers to assess changes in organizational readiness outcomes throughout the study. The survey includes validated measures as well as “home-grown” items about specific activities, such as whether providers prescribed a medication and any barriers to doing so. Surveys are web-based, or, for providers with less access to email, through in-person, paper and pencil surveys.

##### Measures

We measure organizational readiness using outcomes for implementation research recommended by Proctor et al. (2011) [58]. We will evaluate the following outcomes specifically related to our organizational readiness intervention: (1) *Acceptability*. Acceptability refers to satisfaction among implementation stakeholders with the complexity of an EBP or new practice (such as the ICC intervention) and relative advantage over current practices [58]. To assess acceptability we adapted items for the staff survey from Moore and Benbasat’s [68] validated instrument which maps onto parallel elements of Roger’s elements of successful diffusion (i.e., complexity, relative ease of use) [69]. An example of these items is: *Prescribing extended-release injectable naltrexone for patients with alcohol use disorders at this clinic would be relatively easy to do*. We also include locally developed items in the survey to capture barriers to use as well as items from the National Center for Addiction and Substance Abuse’s (CASA) National Survey of Primary Care Physicians and Patients on Substance Abuse [70] that capture providers’ opinions about the effectiveness of OAUD EBP, as well whether providers find it difficult to discuss OAUD with their patients. We ask specific questions about acceptability in the focus groups and interviews, such as: *How easy or difficult would it be for providers to prescribe and administer extended-release injectable naltrexone? What are some of the barriers? What changes would have to be made to make it more acceptable?* (2) *Appropriateness*. This refers to the “perceived fit, relevance, or compatibility” [58] of the EBP and ICC intervention in the clinic. We have adapted items from Moore and Benbasat [68] that measure compatibility of EBP and the ICC intervention with the clinic and with current work style (also an element of Roger’s diffusion theory) [69]. This includes items such as: *I think the ICC intervention will fit with the way I like to work*. We also include items from the “Substance Abuse Attitudes Survey (SAAS)” to measure changes in provider attitudes about people with substance abuse disorders [71]. The focus groups and interviews also capture reasons why the EBP or ICC intervention may or may not be perceived as compatible with work style and with other approaches to managing patients with OAUD or introducing new practices into the clinic. (3) *Intent to adopt the EBP*. We assess intention to adopt EBP in several ways. First, we incorporate into the survey the *EBP Attitude Scale (EBPAS)*. The EBPAS is a brief (15-item), valid, reliable measure that assesses general attitudes toward adoption of new clinical practices [72]. Next, we use items from Moore and Bensabet’s scale that measure elements associated with successful adoption of new EBP [68]. To measure intention or willingness to adopt, we use the “demonstrability” scale, which asks questions such as “*I believe I can communicate to others the consequences of using extended release injectable naltrexone*.” Finally, we ask questions in the focus groups and interviews about intent to adopt. (4) *Feasibility*. Feasibility is the actual fit, utility, and suitability of a program within an organization: the practicability [58]. We assess feasibility retrospectively by asking participants in focus groups and interviews whether the EBP and ICC intervention were successfully implemented and whether poor resources, training, or other barriers impeded use. We also ask about feasibility in the provider survey using items from CASA’s National Survey of Primary Care Physicians and Patients on Substance Abuse that capture how prepared providers feel they are to treat patients with SUD [70]. (5) *Adapted EBP and intervention protocols*. Our final measure of readiness is finalized, adapted protocols for each of the three EBP and the ICC service delivery intervention, which describe how the EBP fit into the clinic workflow. Adapted, finalized protocols are key to ensuring that staff can visualize how the EBP and ICC intervention will be implemented.

##### Analysis plan

The semi-structured interview and focus group data will be analyzed to identify key facilitators and barriers to implementation using classic content analyses. Our quantitative analysis of survey items will consist of pre-post, one-way repeated measures ANOVA comparisons of survey responses between pre- and all post-intervention periods.

#### Service delivery intervention

To examine the effect of our service delivery intervention, we examine service system and patient functioning outcomes.

##### Procedures

We use a combination of administrative records, patient interviews and staff surveys to evaluate service system and patient outcomes, as follows: 1) *Administrative records*. We collect three administrative files every 6 months—appointments (all appointments scheduled whether or not they were kept), encounters (including medical and therapy visit reasons and diagnoses), and medication orders. 2) *Patient interviews*. The patient interview contains an assessment of SUD diagnoses, substance use frequency and quantity, consequences related to use, and health and mental health functioning items. We administer patient interviews at baseline and 6 months after enrollment. 3) *Staff surveys*. Staff surveys are described above.

##### Service system measures

We are analyzing five service system outcomes: (1) *Patient-centered, collaborative SUD care*. We measure patient experiences using a locally developed measure based on the validated Patient Assessment of Chronic Illness Care (PACIC) [73]. Provider perceptions of collaborative SUD care are measured using the Assessment of Chronic Illness Care (ACIC) [74]. (2) *Patient utilization of SUD services*. This refers to patient linkage to and usage of appropriate treatment. We measure this by examining clinic administrative records that capture all patient encounter dates, types and providers, and by patient self-report of clinic services on the follow-up survey. (3) *Patient utilization of health care services*. This refers to utilization of emergency department and health care services. We measure this by examining clinic administrative records of clinic health care visits and by patient self-report of emergency department visits. (4) *Provider adoption of EBP*. This is a measure of provider use of the EBP (either of the medications or the brief therapy). Although adoption is sometimes thought of as an implementation (or readiness) outcome, we include it with service system outcomes because we believe that both interventions—organizational readiness and service delivery—are needed for adoption of EBP. We measure adoption by examining administrative records for prescription of either medication or use of the therapy and by asking providers to self-report use of the EBP in the survey. (5) *Sustainability of EBP*. This is the extent to which the three EBP are still being utilized during year 5 of the study. Following the end of patient enrollment in the RCT, we will continue to monitor clinic practices to examine whether the EBPs are still being utilized following the end of the RCT.

##### Patient outcomes

We are examining four primary patient outcomes. Patient outcomes are: (1) *Changes in quantity and frequency of substance use*. We measure this using the Timeline Follow-Back (TFLB), a validated instrument that uses a calendar to facilitate recall of substance use over the past 30 days [75]. (2) *Consequences of substance use*. To assess consequences, we use the Shortened Inventory of Problems Alcohol and Drugs (SIP-AD), a validated instrument that assesses consequences related to substance use in the past 90 days [76]. (3) *Functioning*. We assess overall health functioning with the SF12 version 2, four-week recall [77]. We use the Patient Health Questionnaire-9 (PHQ-9) [78] to assess depressive symptoms and the generalized anxiety disorder (GAD) [79] to assess symptoms of anxiety. (4) *Satisfaction with SUD care*. We use an adapted standardized patient satisfaction survey [80] to assess patient satisfaction with SUD services at the clinic.

##### Analysis plan

Our quantitative analysis of provider survey items and administrative data related to service system outcomes will consist of pre-post, one-way repeated measures ANOVA comparisons of survey responses between pre- and all post-intervention periods. To analyze patient-level outcomes, we use an intent-to-treat approach. We will first conduct a bivariate analysis to estimate the uncontrolled association between being in the ICC group and outcome. In addition, even though our design randomly assigns patients, we will assess any possible imbalance in covariates between ICC and SAU groups including age, gender, race/ethnicity, and education that affect the impact of the ICC intervention on the outcomes. In cases where observed imbalances are attributable to sample attrition, we will correct for potential bias due to attrition at follow-up using response weights. In addition, characteristics related to an outcome at a conservative significance level of α = 0.2 will be considered covariates in a multivariate analysis for reduction of bias if imbalanced or for efficiency gains. For the multivariate analyses, we will infer about the impact of ICC on an outcome by fitting hierarchical models using SAS Proc Mixed, R LME4, and Winbugs. These models take into account the multi-level structure of the data: two repeated measures over time (baseline and 6 months) nested within patient and patients nested within clinics. Sensitivity analyses will be conducted testing model functional forms, the covariates to be used, and the impact of influential outliers in the analyses results. For outcomes assessed only at month 6 (e.g., treatment satisfaction), we will use cross-sectional analyses (such as linear and logistic regression) to estimate the effect of ICC relative to SAU. This study was designed to estimate sufficient effect sizes that can be detected with a power of at least 80% when comparing the outcomes of patients randomly assigned to the two conditions in an end-status analysis at month 6 for a 5% significance level. For continuous outcomes, the study will be able to detect effect sizes of about 0.30–0.32 standard deviations. These are the kind of effects that can be expected for an intervention like ICC [42]. For dichotomous outcomes, we will be able to detect a difference of 13–14 percentage points under the assumption that the SAU group has a 15% rate of receiving the outcome.

### Trial status

The RCT is currently in month 11 of 18 planned months of active enrollment and data collection.

## Discussion

The present study responds to several critical gaps in health care services for people with SUD—the need for greater access to SUD treatment, the need for more evidence to support the growing emphasis on collaborative, integrated care for SUD in primary care settings, and the call for broader dissemination and adoption of evidence-based treatments for SUD in general and in medical settings in particular. To meet these diverse and complex needs, we designed a multi-level study that (1) combines well-studied implementation tools into an intervention to increase organizational readiness to adopt and sustain SUD EBPs in primary care and (2) tests the effectiveness of a service delivery intervention (ICC) on service system and patient outcomes related to SUD services.

Our hybrid type 2 design [59] allows us to support and study important organizational changes thought to be critical for the adoption and sustainability of new practices and to add what we believe is a necessary component of integrating SUD EBP into primary care—a service delivery intervention tailored to meet clinic specifications and the complex needs of patients with SUD treated in these settings. The study’s unique design takes into account the complexity of introducing new EBPs into a clinical setting, the barriers to integrating SUD EBP into primary care, and the chronic nature of SUD and the corresponding complex needs of SUD patients. We believe that our 18-month organizational readiness phase, starting with preparing the organization for SUD EBP delivery and ending with a pilot phase to ensure that the ICC intervention (i.e., service delivery system intervention) and EBP protocols fit the environment will ensure greater organizational readiness and thus greater likelihood of adoption and sustainability. We believe our multi-level approach—addressing organizational change plus SUD-specific service delivery—is necessary for adoption and sustainability of SUD EBP in primary care. The organizational readiness outcomes will allow us to assess whether our organizational readiness intervention improves provider perceptions of and intention to adopt the EBP while the service delivery intervention will help determine whether the ICC delivery system improves quality of care and patient outcomes compared to service delivery as usual.

Despite the study’s strengths, there are some limitations. One limitation is the lack of provider randomization to test the effects of ICC on provider outcomes. This was determined to be infeasible, due to potential contamination across study conditions and lack of provider and patient support for asking patients to switch providers to match their study condition. Additionally, because we are testing the combined impact of the organizational readiness intervention with the ICC intervention, we will not be able to draw conclusions about the unique contribution of either intervention on EBP implementation, sustainability, or patient outcomes. Moreover, both the organizational readiness and the ICC interventions are complex, containing multiple elements. We will be unable to tease apart the impact of particular elements of the interventions (e.g., the effect of the decision support system from the self-management support) on outcomes. Given the emphasis on examining two complex interventions simultaneously, we elected to examine them initially in one FQHC serving a diverse population in a large metropolitan area in California. It is important to note that this occurred during a time of rapid health care reform especially in California, a state that was an early adopter of Medicaid expansion. We will not know whether our study results will be applicable to other FQHCs or in other geographical locations.

## Additional file

Additional file 1:
**Integrated collaborative care intervention (ICC).** This file contains a detailed description of the elements of the ICC intervention.
