# Supplementary material for: An organizational readiness intervention and randomized controlled trial to test strategies for implementing substance use disorder treatment into primary care: SUMMIT study protocol
Source: Implement Sci. 2015 May 8;10:66. doi: 10.1186/s13012-015-0256-7 (PMC4432875; doi:10.1186/s13012-015-0256-7)
Supplement: Additional file 1: — Integrated collaborative care intervention (ICC). This file contains a detailed description of the elements of the ICC intervention. [file 13012_2015_256_MOESM1_ESM.docx]

**Integrated Collaborative Care Intervention (ICC)**

1. **Delivery System Redesign.** System redesign involves care coordination; an intake session with a behavioral health therapist; redefining staff roles and delineating clear clinical flow for SUD patients; expanding therapy visit-time for SUD patients; and establishing measurement-based care, that is, care that is based on regular monitoring of patients on key outcomes. Each of these is further described below.
   1. **Care Coordinators (CC’s) schedule and organize patient visits.** Upon enrollment in the study, ICC patients receive a warm handoff to a CC, who conducts a 5-10-minute MI-based induction protocol designed to encourage patients to attend an intake session with a behavioral health therapist. CC’s also track patient mental health and medical visits, make reminder and follow-up calls, and attend weekly meetings with behavioral health therapists to review the treatment plan for each patient. The CC’s in the present study are paraprofessionals who also have other responsibilities at the clinic.^[[1]](#footnote-1)^ There is one CC at each site.
   2. **Intake Sessions encourage treatment and self-management.** ICC patients are encouraged by the CC’s to attend an MI-based intake session with behavioral health therapists who are designated as “ICC” therapists. The intake session involves assessment of the patient’s SUD using the ASSIST[84], followed by presentation of a menu of treatment options that is personalized and based on the patient’s level of substance use and need and readiness for treatment. The session is designed to help patients determine their own readiness for treatment and to create an initial, personalized “change plan.” The change plan might include seeing a medical provider to discuss one of the evidence-based medications (if appropriate); receiving a six-session, MI-based behavioral intervention from the behavioral health therapist, receiving referral to local self-help meetings, or it might consist of watchful waiting or the patient opting out of current treatment. Patients might also be referred to a higher level of care, such as inpatient, medically assisted detoxification other inpatient care or intensive outpatient care, if one is warranted.
   3. **Extended visit times to allow providers adequate time for a full SUD assessment.** Therapy visit times are extended from the usual 60-minutes to 90-minutes for ICC intake appointments.
   4. **Measurement-based care allows for targeted disease management**. CC’s monitor patient progress using validated outcome scales and urine toxicology screens. At each visit a graphical report is generated and reviewed by the therapist or medical provider to assess patient progress and modify the treatment as necessary.
2. **Clinical Information Systems.**  To facilitate interdisciplinary communication and coordination, a specialized SUD patient registry and care coordination system alerts CC’s to make reminder and follow-up calls and tracks clinical outcomes such as substance use severity and consequences related to use. A designated folder in the clinic electronic medical record (EMR) contains patient progress reports from the registry, lab results, and therapist notes.
3. **Decision support.** Behavioral health therapists treating ICC patients (the “ICC therapists”) receive weekly supervision from an expert on the MI-based EBP and have unlimited access through email and telephone to this expert and to an addiction medicine specialist. .
4. **Patient Self-Management Support.** To support patients and build their confidence in managing their SUD, CC’s and therapists are trained to review educational and self-management materials on alcohol or opioid abuse upon entering the study.
5. **Community Linkage.** ICC patients receive referral to community support groups as well as referrals to higher levels of care, if needed. ICC patients requiring medically-assisted detoxification prior to receiving XTR-NTX receive expedited entry into a community detoxification facility.

1. In some collaborative care models, CC’s are licensed behavioral health professionals who serve the dual role of administrative coordinator and therapist. [↑](#footnote-ref-1)
